# Transcriptional Regulation of CYP2B6 Expression by Hepatocyte Nuclear Factor 3β in Human Liver Cells

**DOI:** 10.1371/journal.pone.0150587

**Published:** 2016-03-01

**Authors:** Linhao Li, Daochuan Li, Scott Heyward, Hongbing Wang

**Affiliations:** 1 Department of Pharmaceutical Sciences, School of Pharmacy, University of Maryland at Baltimore, 20 Penn Street, Baltimore, Maryland 21201, United States of America; 2 Bioreclamation, IVT, 1450 Rolling Road, Baltimore, Maryland 21227, United States of America; Nihon University School of Medicine, JAPAN

## Abstract

CYP2B6 plays an increasingly important role in xenobiotic metabolism and detoxification. The constitutive androstane receptor (CAR) and the pregnane X receptor (PXR) have been established as predominant regulators for the inductive expression of *CYP2B6* gene in human liver. However, there are dramatic interindividual variabilities in CYP2B6 expression that cannot be fully explained by the CAR/PXR-based modulation alone. Here, we show that expression level of CYP2B6 was correlated with that of hepatocyte nuclear factor 3β (HNF3β) in human primary hepatocytes prepared from 35 liver donors. Utilizing recombinant virus-mediated overexpression or knockdown of HNF3β in HepG2 cells, as well as constructs containing serial deletion and site-directed mutation of HNF3β binding motifs in CYP2B6 luciferase reporter assays, we demonstrated that the presence or lack of HNF3β expression markedly correlated with *CYP2B6* gene expression and its promoter activity. Novel enhancer modules of HNF3β located upstream of the *CYP2B6* gene transcription start site were identified and functionally validated as key elements governing HNF3β-mediated CYP2B6 expression. Chromatin immunoprecipitation assays in human primary hepatocytes and surface plasmon resonance binding affinity experiments confirmed the essential role of these enhancers in the recruitment of HNF3β to the promoter of *CYP2B6* gene. Overall, these findings indicate that HNF3β represents a new liver enriched transcription factor that is involved in the transcription of *CYP2B6* gene and contributes to the large interindividual variations of CYP2B6 expression in human population.

## Introduction

Although traditionally thought to be of minor significance in pharmacology and toxicology [1], the clinical importance of CYP2B6 was recently established with the identification of increasing numbers of CYP2B6 substrates, including clinically important drugs such as the anticancer agents, cyclophosphamide, ifosfamide [2, 3] and tamoxifen [4], the anti-retrovirals efavirenz and nevirapine [5, 6], the anesthetics ketamine and propofol [7, 8], and the central nervous system-active bupropion and methadone [9, 10]. It was estimated that CYP2B6 is involved in the metabolism of nearly 25% of drugs on the market [11]. Marked intra- and inter-individual variation of CYP2B6 expression and activity have been described in the literature and were considered to be attributed mainly to the highly-inducible and polymorphic nature of this gene [12–14]. Many drugs and environmental chemicals can alter the expression of CYP2B6, potentially leading to clinically significant drug-drug interactions. Given the growing importance of CYP2B6 in drug metabolism, the need for a better understanding of the molecular mechanisms governing *CYP2B6* gene expression is evident.

Our understanding of mechanisms underlying transcriptional regulation of CYP2B6 expression has grown substantially during the past two decades, and it has been well-established that induction of CYP2B6 expression by xenobiotics is mediated primarily by the constitutive androstane receptor (CAR, NR1i3) and the pregnane X receptor (PXR, NR1i2) through interactions with phenobarbital responsive enhancer module (PBREM) and xenobiotic responsive module (XREM) located in the distal region of CYP2B6 promoter [15–17]. Nevertheless, large interindividual variability in the expression of CYP2B6 cannot be entirely explained by this simplified CAR/PXR-based model. Activation of CAR and PXR is essential but not sufficient for the optimal regulation of *CYP2B6* gene transcription. Ectopic expression of CAR or PXR alone failed to fully restore the basal and inductive expression of CYP2B6 in non-hepatic cells or hepatoma cell lines that express extremely low levels of liver enriched transcription factors (LETFs) [18]. Several previous reports including our own data demonstrated that expression of *CYP2B6* gene can be influenced by interactions between CAR/PXR and LETFs such as the hepatic nuclear factor 4α (HNF4α) and CCAAT/enhancer-binding protein α (C/EBPα) [19–21], suggesting hepatic factors other than CAR and PXR contribute to the large individual variations in CYP2B6 expression.

The hepatocyte nuclear factor 3β (HNF3β), also known as forkhead box protein A2 (FOXA2), is a DNA-binding protein that is encoded by the *FOXA2* gene in human, and plays a pivotal role in the regulation of metabolism-related gene expression in the liver and pancreas [22]. As a hepatic transcription factor, HNF3β influences the expression of numerous genes involved in energy metabolism, bile acid homeostasis, drug metabolism and transport by interacting with other LETFs such as HNF1α and HNF4α, and nuclear receptors such as the glucocorticoid receptor and PXR [23–25]. Disruption of HNF3β binding sites located in the promoter of glucose-6-phosphatase or CYP3A4 repressed the transcriptional activity of each respective gene [26, 27]. Recently, Lamba et al., reported that hepatic expression of CYP3A4 was positively correlated with that of HNF3β [28]. Given the pleotropic roles of HNF3β in hepatic gene regulation and the known transcriptional cross-talk between CYP2B6 and CYP3A4, we hypothesized that HNF3β may play a role in the transcription of *CYP2B6* gene and contribute to the observed large inter- and intra-individual variations in CYP2B6 expression.

In the current study, we provide experimental evidence to demonstrate that expression of CYP2B6 is closely associated with the expression of HNF3β in human primary hepatocytes (HPH). Overexpression of HNF3β enhanced expression and promoter activity of CYP2B6, while knockdown of HNF3β significantly repressed CYP2B6 expression in HepG2 cells. Utilizing *in silico* analysis, chromatin immunoprecipitation (ChIP) and surface plasmon resonance (SPR) binding affinity assays, characterization of the CYP2B6 promoter revealed two functional enhancer modules that are responsible for HNF3β-mediated CYP2B6 transcription.

## Material and Methods

### Reagents

Phenobarbital (PB) and 6-(4-Chlorophenyl) imidazo[2,1-b][1,3]thiazole-5-carbaldehyde-O-(3,4-dichlorobenzyl) oxime (CITCO) were purchased from Sigma-Aldrich (St. Louis, MO). Oligonucleotide primers were synthesized by Integrated DNA Technologies, Inc. (Coralville, IA). The Dual-Luciferase Reporter Assay System was purchased through Promega (Madison, WI). Antibodies against CYP2B6 and HNF3β were from Santa Cruz (Dallas, TX). β-Actin antibody was from Sigma-Aldrich. Matrigel, insulin, and ITS^+^ (insulin/transferrin/selenium) were obtained from BD Biosciences (Bedford, MA). Other cell culture reagents were purchased from Life Technologies (Grand Island, NY) or Sigma-Aldrich.

### Plasmid Construction

Luciferase reporter plasmids containing -300bp, -387bp, -600bp, -1kb, -1.4kb and -2kb fragments of the CYP2B6 promoter region were PCR-amplified by using forward primers: 5’- GGGGTACCTAGACATACATATACCCAC-3’, 5’- GGGGTACCCATACAGGGATGCAAGCAG-3’, 5’- GGGGTACCGGGATTACAGGTGTGAGC-3’, 5’- GGGGTACCTCAGCATCTGCAGGCTTC-3’, 5’- GGGGTACCACACACCTGGAGCTCAAG-3’, 5’- GGGGTACCGGACAATGTAGCCCCAACCC-3’ and the same reverse primer: 5’- AGTCTACTCGAGCTGCACCCTGCTGCAGCCT-3’. The PCR products were sub-cloned into the KpnI and XhoI sites of pGL3-basic vector, resulting in constructs termed 2B6-300bp, 2B6-387bp, 2B6-600bp, 2B6-1kb, 2B6-1.4kb and 2B6-2kb, and the correct orientation was verified by sequencing. The pCR3-hCAR, pMEX-C/EBPα, pcDNA3.1/HNF3β expression vectors and the CYP2B6 reporter constructs (2B6-1.6kb and 2B6-1.8kb) were obtained or generated as described previously [15, 17, 20]. pRL-TK was used as an internal control.

### Site-directed Mutagenesis

Site-directed mutagenesis was performed by PCR, using the 2B6-2kb construct as the template, based on the protocol of the QuickChange Multi Site-Directed Mutagenesis Kit from Stratagene (Santa Clara, CA). Mutated nucleotides in the HNF3β binding sites are underlined for the HNF3β-a-mut: CACTAAGAGTGTACCGCCTGAGTTACTGTGTG, the HNF3β-b-mut: CCCCTTTACAT GTACCAGTCATATAAGCACATAC, and the HNF3β-c-mut: CAAAGCTAAGTACCAGAGTGCA AGCTCACC. The constructs were sequenced to confirm the presence of the mutation(s).

### HPH Cultures

Human hepatocytes were isolated as described previously [29] from human liver specimens obtained from University of Maryland Medical Center with prior approval by the Institutional Review Board at the University of Maryland at Baltimore or obtained from Bioreclamation In Vitro Technologies (Baltimore, MD). Hepatocytes with viability over 90% were seeded at 0.75 × 10^6^ cells/well in 12-well biocoat plates in DMEM supplemented with 5% FBS, 100 U/ml penicillin, 100 μg/ml streptomycin, 4 μg/ml insulin, and 1 μM dexamethasone. After attachment at 37°C in a humidified atmosphere of 5% CO2, hepatocytes were cultured in complete William’s Medium E (WME) and overlaid with Matrigel (0.25mg/ml). Cell culture medium was replaced on a daily basis.

### Real-Time PCR Analysis

Total RNA from human primary hepatocytes was isolated using the RNeasy Mini Kit (Qiagen, Valencia, CA) and reverse transcribed using High Capacity cDNA Archive kit (Applied Biosystems, Foster City, CA) following the manufacturers’ instructions. Real-time PCR assays were performed in 96-well optical plates on an ABI StepOnePlus Real-Time PCR system (Applied Biosystems) with SYBR Green PCR Master Mix (Qiagen). Primer sequences for the CYP2B6, CAR, HNF3β, C/EBPα, HNF4α, and glyceraldehyde-3-phosphate dehydrogenase (GAPDH) are: CYP2B6, 5’-AGACGCCTTCAATCCTGACC-3’ and 5’-CCTTCACCAAGACAAATCCGC-3’; CAR, 5’- GAGCTGAGGAACTGTGTGGTA-3’ and 5’-CTTTTGCTGACTGTTCTCCTGAA-3’; HNF3β, 5’- GGAGCAGCTACTATGCAGAGC-3’ and 5’-CGTGTTCATGCCGTTCATCC-3’; C/EBPα, 5’-TCGGTGGACAAGAACAGCAA-3’ and 5’-TTTCAGGAGGCACCGGAATCT-3’; HNF4α, 5’-CGAAGGTCAAGCTATGAGGACA-3’ and 5’- ATCTGCGATGCTGGCAATCT-3’; and GAPDH,5’-CCCATCACCATCTTCCAG GAG-3’ and 5’-GTTGTCATGGATGACCTTGGC-3’. Fold induction values were calculated according to the equation 2^ΔΔCt^, where ΔCt represents the differences in cycle threshold numbers between the target gene and GAPDH, and ΔΔCt represents the relative change in these differences between control and treatment groups.

### Transient Transfection in HepG2 Cells

HepG2 cells obtained from American Type Culture Collection (Manassas, VA) were transfected with different CYP2B6 reporter constructs in the presence of HNF3β, with or without hCAR and C/EBPα expression vectors using X-tremeGENE 9 DNA Transfection Reagent (Roche Diagnostics Corporation, Indianapolis, IN). Twenty-four hours after transfection, cells were treated with solvent (0.1% DMSO), PB (1mM), or CITCO (1μM) for 24 h. Subsequently, cell lysates were assayed for firefly luciferase activities normalized against the activities of co-transfected Renilla luciferase using Dual-Luciferase Kit (Promega, WI). Data were represented as mean ± S.D. of three individual transfections.

### HNF3β overexpression and knockdown in HepG2 cells

Twenty-four hours after seeding, HepG2 cells were infected with negative control adenovirus or increasing concentrations of adenovirus expressing HNF3β purchased from Vigene Biosciences (Rockville, MD) for 48 h. For HNF3β-RNAi lentivirus, DNA oligonucleotides encoding short hairpin HNF3β RNA (GATCAAGAACATGTCGTCGTACGTGCTCGAGCACGTACGACGACATGTTCTTTTTTTG) [30] was inserted into the BamHI and EcoRI restriction sites of the pGreenPuro™ shRNA expression lentivector from System Biosciences (Mountain View, CA). Lentivirus was prepared as described previously [31]. In HNF3β knockdown assay, HepG2 cells were infected with the lentiviral particles for 96 h. Total RNA and Proteins were prepared for real-time PCR and western blot analysis.

### Western Blot Analysis

Cell homogenate proteins harvested from Ad-HNF3β overexpressing HepG2 cells were resolved on SDS—polyacrylamide gels (12%) and electrophoretically transferred onto blotting membranes. Subsequently, membranes were incubated with antibodies against HNF3β (diluted 1:200), CYP2B6 (diluted 1:200), or β-actin (diluted 1:50,000). Blots were washed and incubated with horseradish peroxidase secondary antibodies, and developed using enhanced chemiluminescence Western blotting detection reagent from GE Healthcare (Pittsburgh, PA).

### CYP2B6 Activity Assay

CYP2B6 metabolic activity assay was performed in adenovirus HNF3β infected HepG2 cells on 24-well plate using P450-Glo ™ CYP2B6 Assay kit (Promega, WI) following the manufacturer’s instruction. In brief, 48 h after HNF3β adenovirus infection, culture medium was completely removed and cells were washed twice with Krebs-Henseleit buffer (BioreclamationIVT, MD), followed by incubation at 37°C for 2 h in 300 μL Krebs-Henseleit buffer containing 3 μM of Luciferin-2B6 (CYP2B6 specific substrate) and 3 mM of salicylamide. Equal volume of Luciferin Detection Reagent was added into each well, and mixed at room temperature for 20 min before luminescence detection (Promega, WI). Data were represented as mean ± S.D. of three individual infections.

### Chromatin Immunoprecipitation Assays

Experiments were performed using a ChIP assay kit according to the manufacturer’s protocol (Millipore Corporation, Billerica, MA). In brief, 1 × 10^6^ cultured human primary hepatocytes were cross-linked with 1% formaldehyde for 10 min at 37°C, washed with ice-cold phosphate buffered saline containing a protease inhibitor cocktail. Nuclear extracts were sonicated. Immunoprecipitation was performed overnight at 4°C using 5μg of anti-HNF3β antibody (Santa Cruz Biotechnology) or normal rabbit IgG (Cell Signaling Technology) followed by precipitation using protein A coupled to agarose beads. After de-crosslinking and protease digestion, DNA fragments were recovered by QIAquick PCR purification kit (QIAGEN). CYP2B6 promoters containing HNF3β-a (distal), HNF3β-c (proximal), and the region between -1.4/-1.6k were amplified by PCR using primers: 5’-TGGACAATGTAGCCCCAACCC-3’ and 5’-GATTGGGTGCTCATTGCAGCC-3’; 5’-CTCATACACATGCAAGGATAC-3’ and 5’-GAGCAAGTGAATGTGTGGG TG-3’; and 5’-GTCAGGCGTAGGATGAGACAG-3’and 5’-TCTTGAGCTCCAGGTGTGTGC-3’. A proximal promoter region of *SULT1E1* gene was used as a negative control as reported previously [32].

### Plasmon Resonance Binding Assay

Recombinant HNF3β protein purchased from Abcam (Cambridge, MA) was covalently linked to the surface of a BIAcore CM5 sensor chip (BR-1005-30, Lot#10222950) by direct immobilization as described previously [33]. Oligonucleotides containing HNF3β-a (AGAGTGTAAAGACTGAG), HNF3β-b (TACATGTAAAAATCATA) and HNF3β-c (GCTAAGTAAAAAAGTGC) were used as analytes. The binding assay was performed by injecting 60 μl each of the oligonucleotides at 10 μM in 10 mM Hepes, pH 7.4, containing 150 mM NaCl, 3 mM EDTA, and 0.005% P-20 at the flow rate of 30 μl per minute at 25°C. The association and dissociation between analytes and HNF3β protein were recorded respectively by SPR with a Biacore 3000 (GE Healthcare, Piscataway, NJ) following the manufacturer’s instructions. Sensorgrams of the interaction generated by the instrument were analyzed by the software BIAeval 3.2.

### General data analysis

All data represent at least three independent experiments and were expressed as mean ± S.D. Statistical significance was determined using one-way analysis of variance followed by post-hoc Dunnett’s test or Student’s t test where appropriate. Statistical significance was set at *p* < 0.05 or *p* < 0.01. Linear regression was analyzed using Pearson’s Correlation Coefficient (JMP 7.0; SAS, NC).

## Results

### Correlation of CYP2B6 and HNF3β expression in HPH

**Fig 1:**
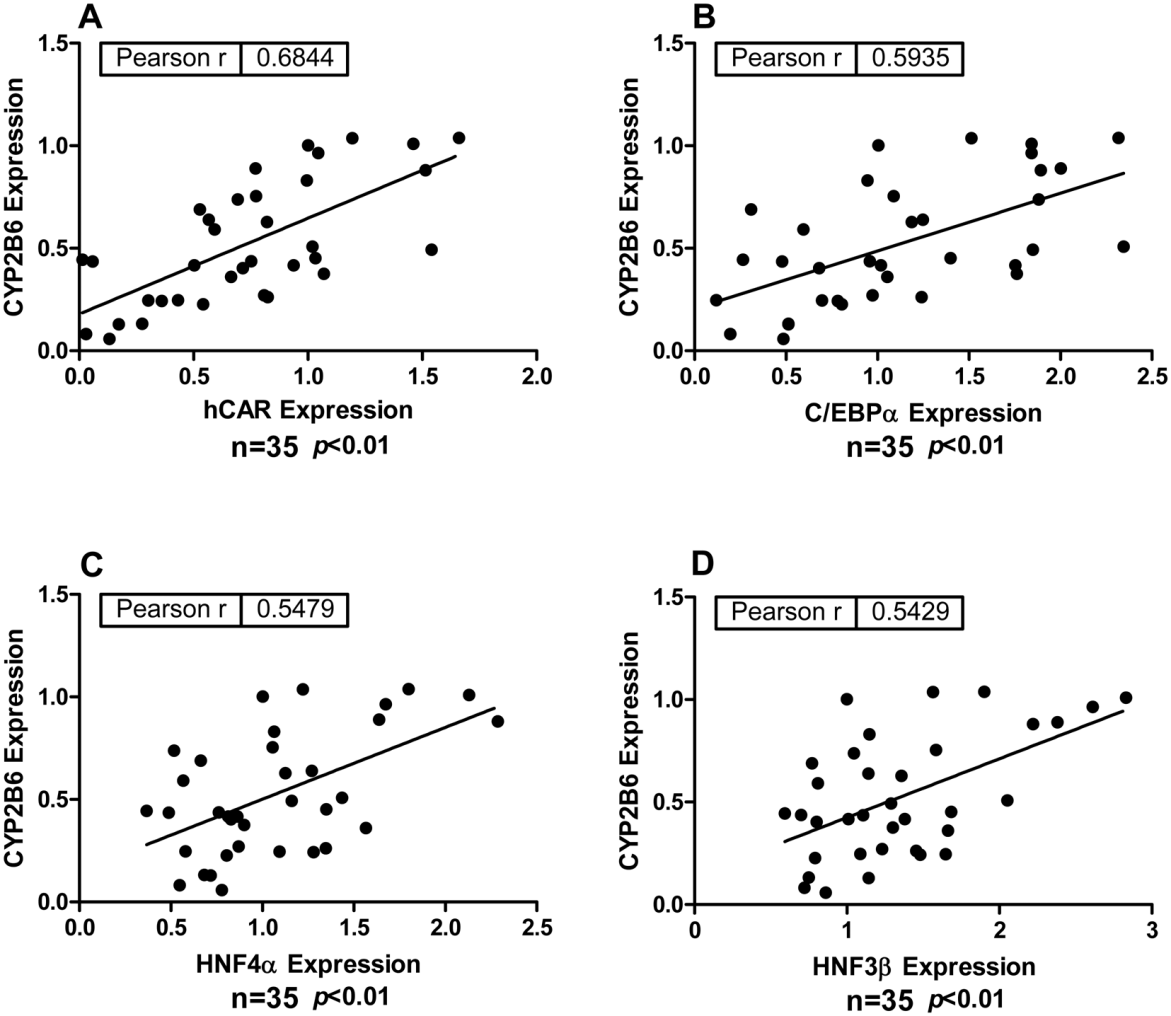
Correlation between CYP2B6 and HNF3β expression in HPH. Total RNA was extracted from HPHs prepared from 35 human liver donors. Expression levels of CYP2B6, hCAR, C/EBPα, HNF4α, HNF3β were measured using real-time RT-PCR assays as detailed in the *Materials and Methods*. Relative gene expression levels from all donors were normalized against a randomly selected single donor. Leaner regression between CYP2B6 and one of these hepatic transcriptional factors was analyzed individually using Pearson’s Correlation Coefficient (JMP 7.0; SAS, NC).

Correlation between the expression of a number of hepatic transcription factors and CYP2B6 was initially evaluated in a collection of HPH prepared from 35 human liver donors. Basal mRNA expression of these genes in hepatocytes was determined by real-time PCR assays. As expected, positive correlations were observed between the expression of CYP2B6 and that of CAR (R = 0.6844; *p* < 0.01), C/EBPα (R = 0.5935; *p* < 0.01), and HNF4α (R = 0.5479; *p* < 0.01), respectively (Fig 1A–1C). Interestingly, the abundance of CYP2B6 in these donors was also significantly correlated with the expression of HNF3β (R = 0.5429; *p* < 0.01) (Fig 1D). These results indicate that besides the known role of CAR, HNF4α and C/EBPα in CYP2B6 expression, HNF3β may represent another LETF that contributes to the transcription of *CYP2B6* gene in human liver.

### Ectopic expression of HNF3β alters CYP2B6 expression and activity in HepG2 cells

**Fig 2:**
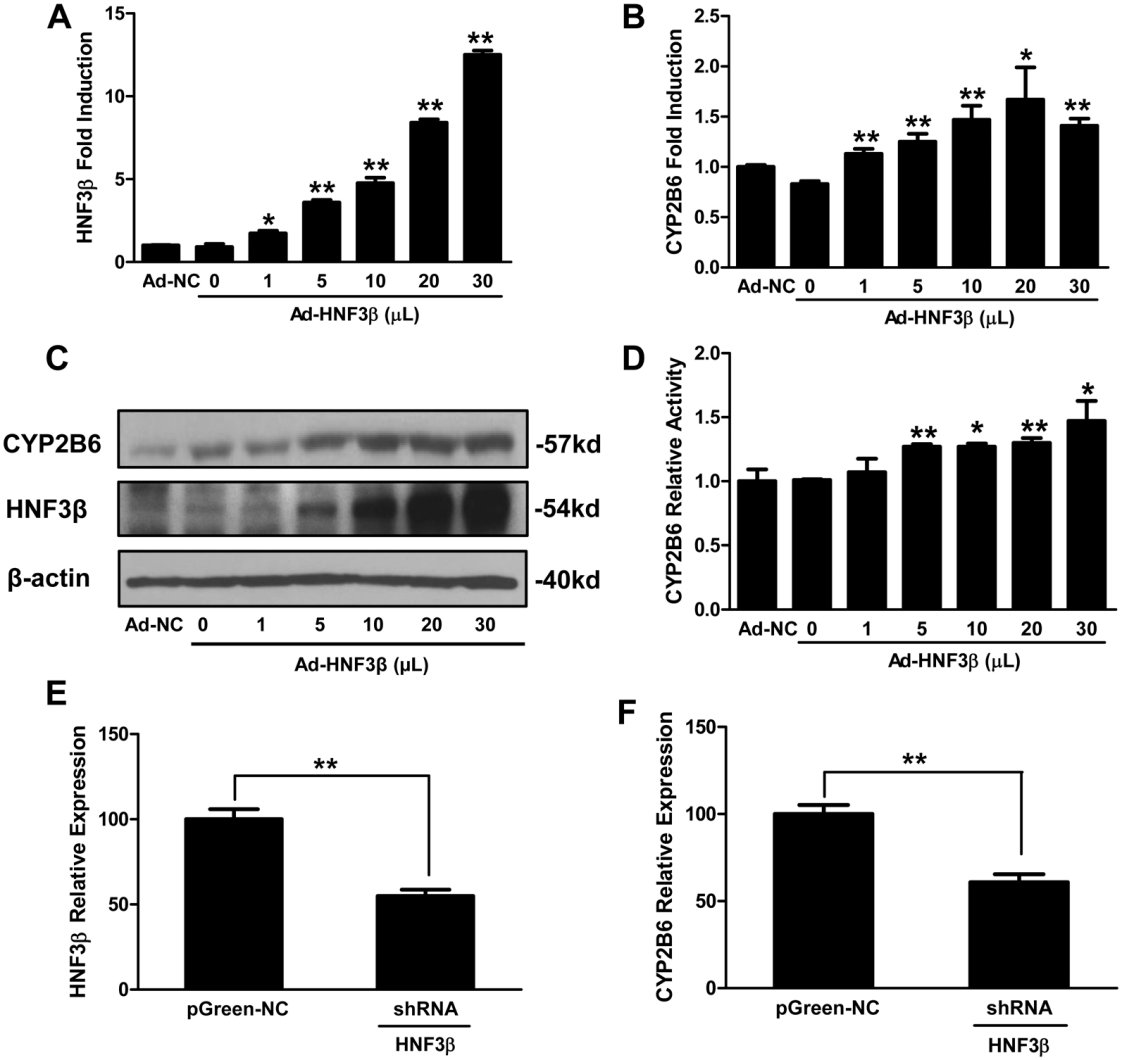
Effects of HNF3β on the expression and activity of CYP2B6 in HepG2 cells. HepG2 cells were infected with Negative Control Adenovirus (Ad-NC) or various amounts of adenovirus expressing HNF3β (Ad-HNF3β) for 48 h. Expression of HNF3β mRNA (A), CYP2B6 mRNA (B), and their proteins (C) were measured using real-time PCR and Western blotting assays, respectively. CYP2B6 enzymatic activity (D) was detected using P450-Glo^™^ CYP2B6 Assay kit (Promega). In separate experiments, HepG2 cells were infected with pGreen Negtive Control Lentivirus (pGreen-NC) or HNF3β-RNAi lentivirus (HNF3β-shRNA) for 96 h before measuring the mRNA expression of HNF3β (E) and CYP2B6 (F) by real-time PCR. Results are expressed as the mean ± S.D. (n = 3). (*P <0.*05*, **P <0.*01*).

To further investigate the effects of HNF3β on CYP2B6 expression, adenovirus-driven HNF3β was used to ectopically over-express HNF3β in HepG2 cells. As shown in Fig 2A and 2B, mRNA expression of HNF3β by virus infection was associated with enhanced expression of CYP2B6 in HepG2 cells in a concentration-dependent manner. A similar pattern of increased CYP2B6 protein content and enzyme activity was also observed (Fig 2C and 2D). On the other hand, knockdown of HNF3β by lentiviral-shRNA was associated with decreased expression of CYP2B6 in HepG2 cells (Fig 2E and 2F). Together, these results indicate that the intracellular level of HNF3β influences expression of CYP2B6 in HepG2 cells and support the positive correlation between hepatic expression of HNF3β and CYP2B6 observed in human liver donors.

### HNF3β activates the transcriptional activity of a CYP2B6 reporter gene

**Fig 3:**
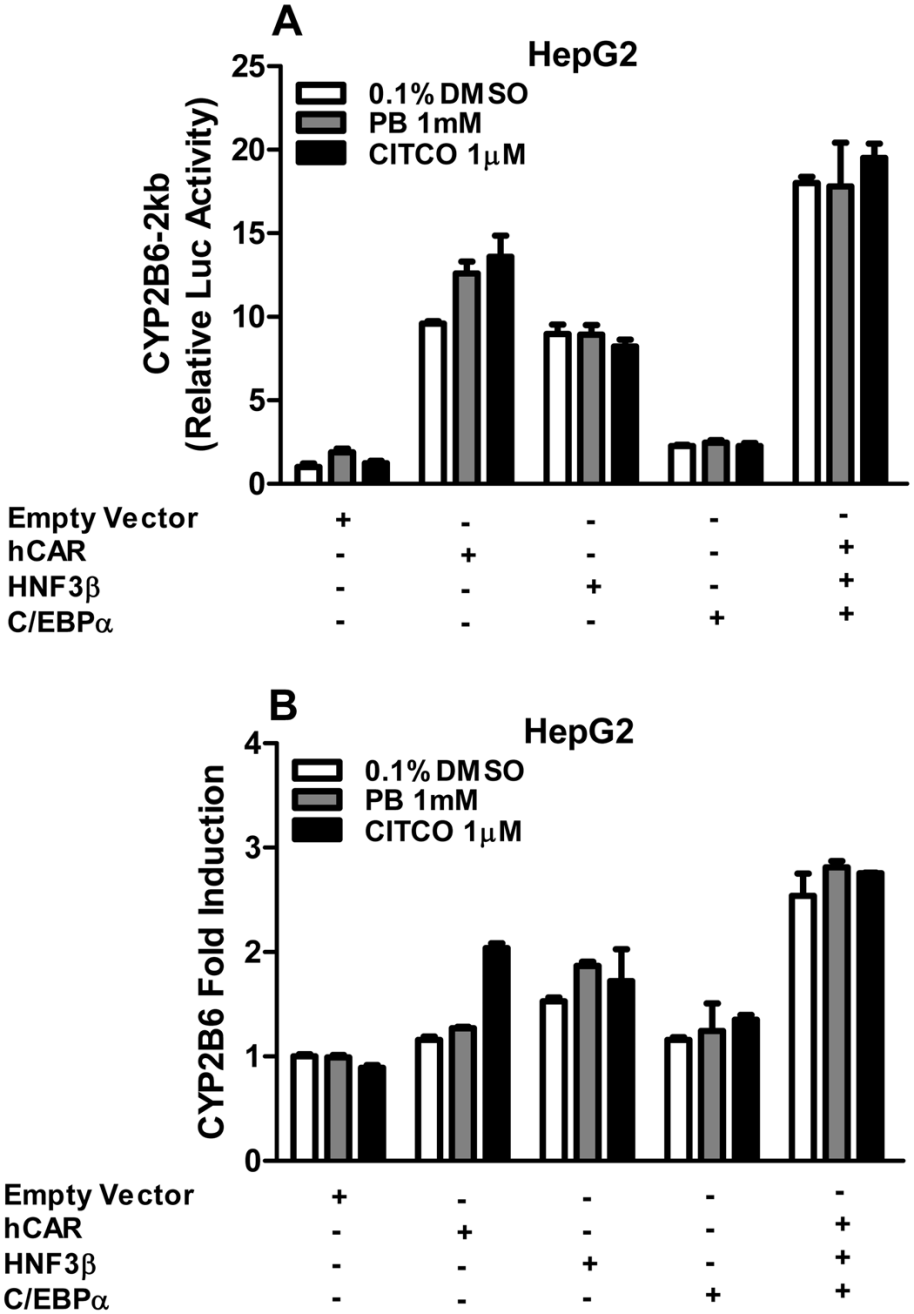
Effects of HNF3β on hCAR-mediated CYP2B6 activation in HepG2 cells. HepG2 cells were co-transfected with expression plasmids of hCAR, HNF3β or C/EBPα in the presence (A) or absence (B) of CYP2B6-2kb reporter construct as detailed in the *Materials and Methods*. Transfected cells were then treated with PB (1mM) and CITCO (1 μM) for 24 h. Dual luciferase activities (A) and CYP2B6 mRNA expression (B) were detected and expressed relative to vehicle control by reporter assay and real-time PCR analysis.

It is well-known that expression of CYP2B6 is predominantly regulated at the transcriptional level. Thus, we next investigated whether HNF3β could alter the transcriptional activity of CYP2B6 via a putative luciferase construct containing the first 2 kb of the CYP2B6 5’-flanking region in a pGL3-Basic reporter vector. This region contains the PBREM regulatory module that responds to the nuclear receptor CAR. Consistent with the notion that CAR is constitutively activated in immortalized cell lines [14], transfection of CAR robustly increased the luciferase activity of CYP2B6-2kb, while additional treatment with PB or CITCO only moderately increased the CYP2B6 luciferase activity (Fig 3A). Notably, transfection of HNF3β dramatically enhanced the activation of the CYP2B6 reporter independent of chemical treatment. Co-transfection of hCAR/HNF3β/C/EBPα/CYP2B6-2kb resulted in an additive increase of the luciferase activity in HepG2 cells. Likewise, co-transfection of hCAR and/or HNF3β/C/EBPα as aforementioned also increased expression of endogenous CYP2B6 mRNA (Fig 3B) in HepG2 cells. Together, these findings suggest that the first 2 kb of the CYP2B6 promoter may contain specific sequences that coordinate HNF3β-mediated CYP2B6 transactivation.

### Identification of HNF3β-Response Elements in CYP2B6 promoter

**Fig 4:**
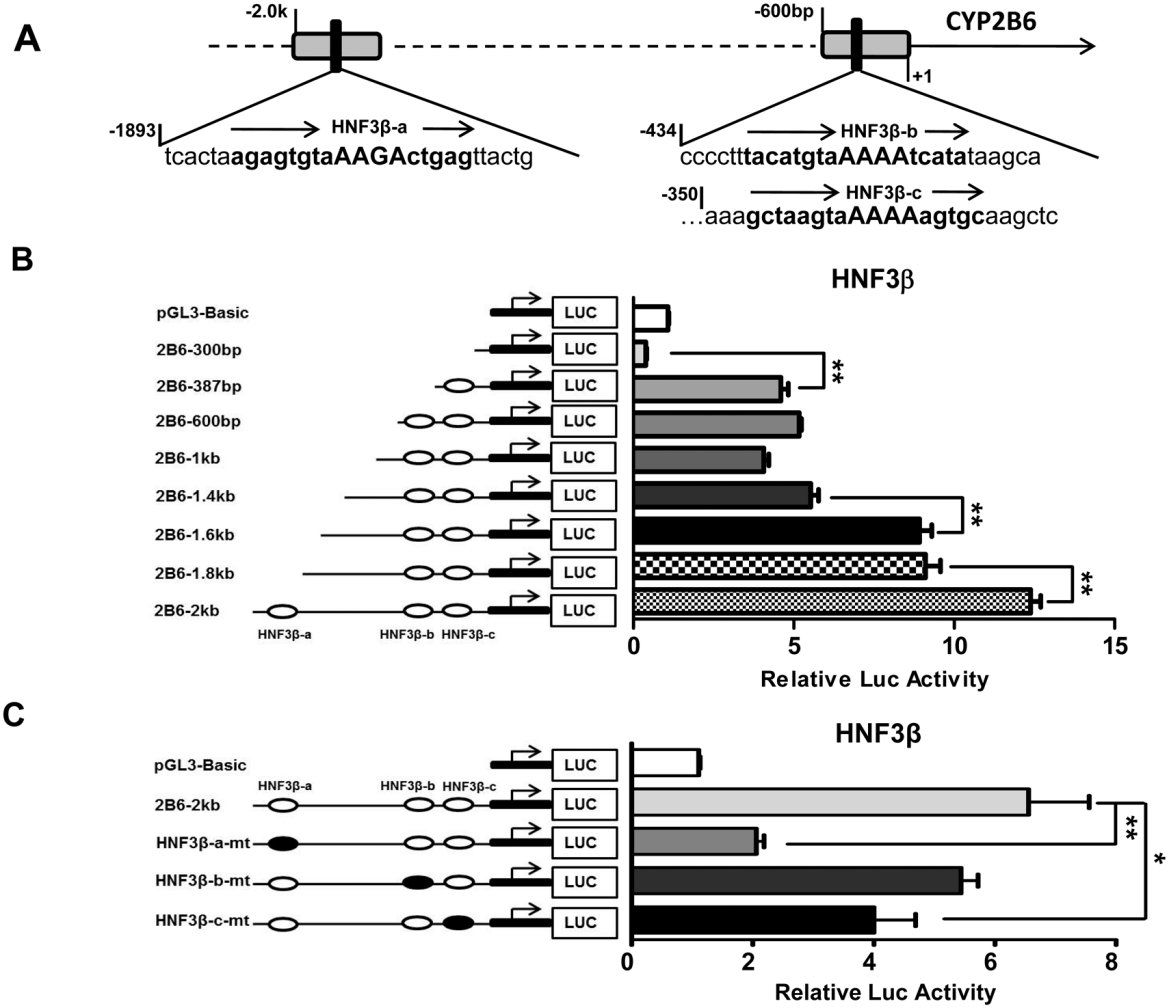
Transactivation of CYP2B6 5’-flanking reporter constructs by HNF3β. A computer-based search for the first 2 kb of CYP2B6 upstream resulted in the identification of three potential HNF3β-responsive elements located between -1893bp and -350bp (A). HepG2 cells were transfected with HNF3β expression vector in the presence of CYP2B6 promoter constructs containing sequential deletion fragments (B) or the CYP2B6-2.0k harboring one of the mutated HNF3β binding sites (C). Forty eight hours post-transfection, luciferase activities were determined and expressed relative to the control (pGL3-Basic). Data represent the mean ± SD. (n = 3). (*, *p <0*.05; **, *p<0*.*01*).

To elucidate the molecular basis underlying HNF3β-mediated CYP2B6 transcription, we carried out an *in silico* analysis of the first 2 kb upstream sequence of the CYP2B6 promoter using the MatInspector release professional program [34]. Two clusters of potential HNF3β binding sites were localized at -1887/-1871bp and -434/-350bp regions. As shown in Fig 4A, three predicted HNF3β responsive motifs in the two clusters were designated as HNF3β-a, HNF3β-b, and HNF3β-c. Serial deletion reporter assays revealed that maximal transactivation of CYP2B6 promoter by HNF3β was achieved with the CYP2B6-2kb construct that contained all predicted enhancers. Deletion of HNF3β-a or HNF3β-c significantly repressed the role of HNF3β in CYP2B6 promoter activation, while elimination of HNF3β-b only exhibited negligible consequence (Fig 4B). In site-directed mutagenesis experiments, transactivation of CYP2B6-luciferase activity through HNF3β was remarkably attenuated by the mutation of HNF3β-a or HNF3β-c, while only moderately affected by HNF3β-b mutation (Fig 4C). Interestingly, although our *in silico* analysis failed to predict a consensus HNF3β binding site between -1.6 kb and -1.4 kb, our luciferase reporter assay showed that deletion of this 200 bp sequence significantly reduced HNF3β-mediated activation of CYP2B6 promoter (Fig 4B), indicating the existence of a functional yet unidentified HNF3β binding site in this region. Together, these data suggest that transcriptional activation of CYP2B6 by HNF3β is mediated through multiple enhancer modules including the newly identified HNF3β binding sites, particularly the HNF3β-a and HNF3β-c motifs.

### Interaction between HNF3β and enhancers identified in the CYP2B6 promoter

**Fig 5:**
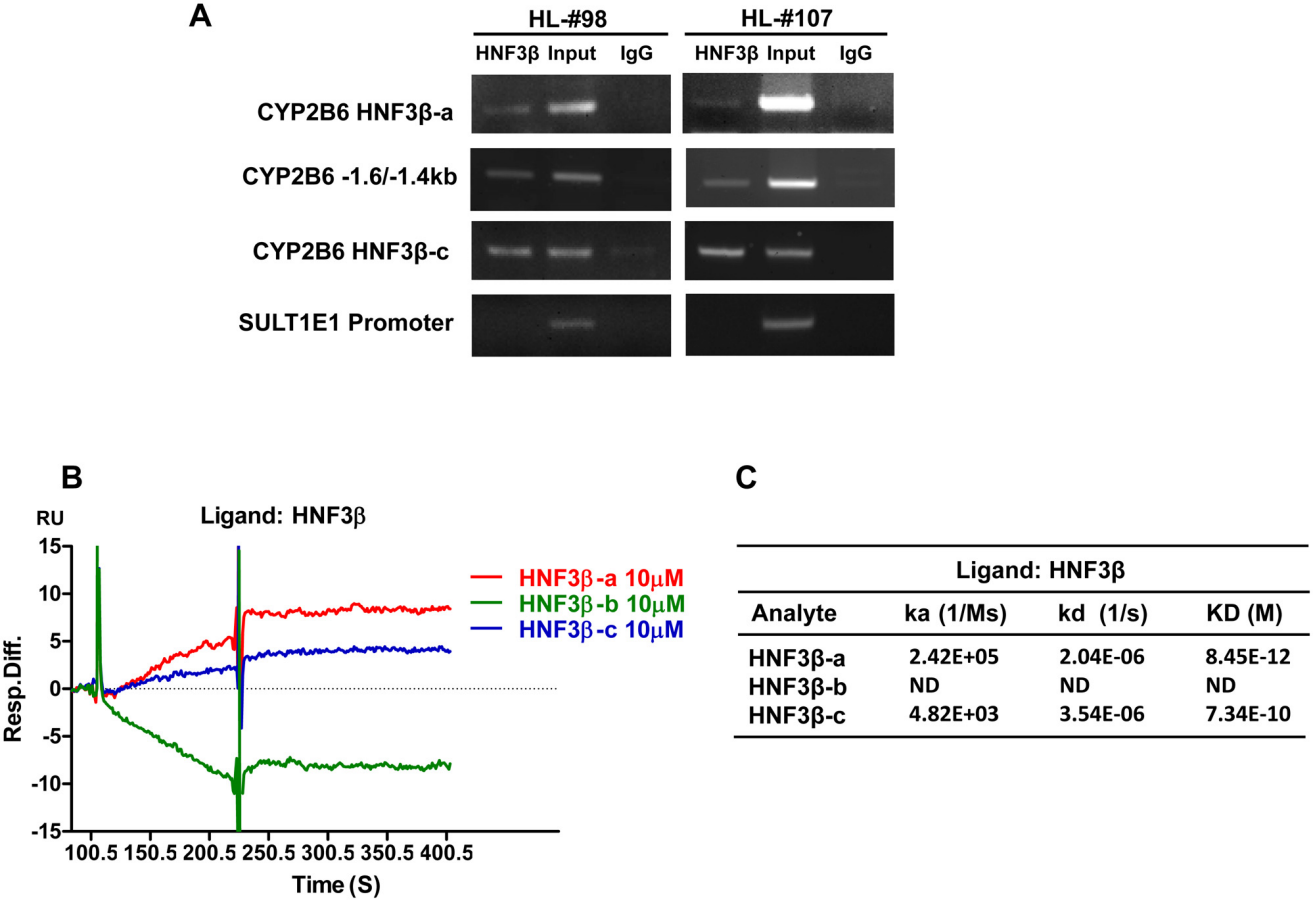
Recruitment of HNF3β to enhancers identified upstream of CYP2B6 promoter. As detailed in the *Materials and Methods*, ChIP assays were used to analyze binding of HNF3β to the HNF3β-a (distal), HNF3β-c (proximal) containing, and the -1.6/-1.4kb regions in cultured HPH (A). After precipitation with HNF3β antibody, de-crosslinked DNA fragments were amplified by PCR. Amplification of the promoter of SULT1E1 was used as negative control as reported previously. In separate experiments, BIACORE SPR affinity assays (B) and (C) were carried out to measure the comparative binding kinetics of CYP2B6 enhancers on HNF3β as described under *Materials and Methods*. Sensorgrams of the interaction generated by the instrument were analyzed by the software BIAeval 3.2.

Potential physiological recruitment of HNF3β to the CYP2B6 promoter was assessed using ChIP assays in cultured HPH from two liver donors (HL#98 and #107). As demonstrated in Fig 5A, endogenous HNF3β protein was efficiently recruited to the distal and proximal promoter regions of CYP2B6 containing HNF3β-a and HNF3β-c, respectively. Lack of binding to the promoter region of SULT1E1 was used as a negative control as reported previously [32]. Notably, our results showed that HNF3β protein was also enriched in the -1.6/-1.4 kb region, further supporting the presence of a functional HNF3β binding site in this region. The binding kinetics between HNF3β and CYP2B6 enhancers were further validated using a SPR binding affinity assay. As shown in Fig 5B, DNA sequences (analytes) containing HNF3β-a or HNF3β-c bind to HNF3β efficiently, while HNF3β-b exhibits undetectable association with HNF3β. Detailed kinetics calculation revealed that HNF3β-a had a higher association rate constant (2.42 × 10^5^ 1/Ms) in comparison to that of HNF3β-c (4.82 × 10^3^ 1/Ms) though both shared similar dissociation rate constants (2.04 ×10^−6^ and 3.54 × 10^−6^ 1/s, respectively). In consequence, HNF3β-a exhibited a higher binding affinity (KD = 8.45 ×10^−12^ M) than HNF3β-c (KD = 7.34 ×10^−10^ M) (Fig 5C). Together, these results indicate that HNF3β can efficiently interact with the CYP2B6 promoter with higher binding affinity to the HNF3β-a than the HNF3β-c motifs, while there is no detectable binding to the HNF3β-b site.

## Discussion

CYP2B6, an inducible cytochrome P450 isoform predominantly present in the liver, exhibits large intra- and inter-individual variations in human populations [13, 35]. Expression of CYP2B6 is controlled by many transcription factors including drug and hormone responsive nuclear receptors such as CAR, PXR, the vitamin D and glucocorticoid receptors, and constitutively activated LETFs including HNF4α and C/EBPα [21, 36–38]. Our current study reveals that HNF3β represents a novel LETF that can up-regulate *CYP2B6* gene expression by recognizing and interacting with multiple enhancer modules including two consensus HNF3β binding sites located at -1887/-1871bp and -347/-331bp upstream of the CYP2B6 transcriptional start site. Site-directed mutation of these responsive elements resulted in significant reduction of the CYP2B6 promoter activity. Moreover, HNF3β protein was efficiently recruited to the CYP2B6 promoter through direct interaction with these enhancer modules.

To date, human CAR and PXR have been recognized as the major transcription factors mediating drug-induced CYP2B6 expression. However, optimal expression of CYP2B6 was only achieved in HPH that maintain physiologically relevant expression of most LETFs. Hepatocyte nuclear factors are a heterogeneous group of evolutionarily conserved transcription factors that are pivotal for the development and maintenance of liver specific features [23]. Positive correlations between expression of CYP2B6 and HNF4α or C/EBPα (also named HNF2) were demonstrated previously in human liver samples [20, 39]. Knockdown of HNF4α expression decreased the mRNA level of CYP2B6, CAR, and PXR in HPH [40], while ectopic co-expression of HNF4α, CAR, and C/EBPα markedly increased *CYP2B6* gene transcription in HepG2 cells [21]. Using HPH from 35 individuals, we found that expression of CYP2B6 was positively associated with the expression of HNF3β, in addition to its known correlation with CAR, HNF4α and C/EBPα. These initial observations led to a more focused investigation on the role of HNF3β in hepatic CYP2B6 transcription.

Regarded as one of the master regulators of hepatocyte differentiation and maturation, HNF3β regulates the expression of numerous hepatic genes by interacting with respective cis-acting binding elements in the promoters of these genes, including the gluconeogenic phosphoenolpyruvate carboxykinase, insulin-like growth factor-binding protein 1, and tyrosine aminotransferase [41–43]. Unlike ligand-activated transcription factors, presence of the HNF3β protein alone in hepatocytes is sufficient to trigger downstream signaling pathways and the abundance of HNF3β protein often correlates well with the expression of its target genes [27, 28]. Additionally, cooperation between LETFs and ligand-activated nuclear receptors can contribute to tissue-specific increase or decrease in expression of nuclear receptor target genes. For instance, overexpression of HNF4α facilitates CAR- and PXR-mediated induction of CYP2B6, CYP3A4, and CYP2C9 [21, 44, 45], while HNF4α-mediated expression of CYP7A1, a key enzyme in cholesterol metabolism and bile acid synthesis, was down-regulated by competing with CAR for binding to the direct repeat 1 (DR1) motif in the promoter of CYP7A1 [46]. We have previously shown that a single nucleotide polymorphism introducing a functional C/EBPα binding site at the proximal region of CYP2B6 promoter synergistically enhanced PXR-mediated induction of this gene [20]. In the current study, we found that overexpression of HNF3β increased the activity of CYP2B6 reporter construct, and the endogenous expression of CYP2B6 mRNA and protein in HepG2 cells. Notably, although overexpression of either CAR or HNF3β increased CYP2B6 promoter activity, their combination only produced additive effects on the transactivation of CYP2B6 in transfected HepG2 cells, suggesting CAR and HNF3β may influence CYP2B6 expression independently rather than collaboratively. Subsequent computer-based analysis revealed the presence of three potential HNF3β binding sites at -1887/-1871bp, -347/-331bp, and -428/-412bp regions of the first 2 kb of the CYP2B6 promoter. Luciferase reporter assays demonstrated that maximal activation of CYP2B6 reporter by HNF3β requires the presence of all three elements. Of importance, serial deletion and mutation assays further revealed the importance of the HNF3β-a and HNF3β-c motifs, while HNF3β-b only exhibited minimal activity. Intriguingly, although no consensus HNF3β binding site(s) was predicted from our *in silico* analysis in the -1.6k to -1.4k region of the CYP2B6 promoter, deletion of this region unexpectedly resulted in a significant reduction of CYP2B6 luciferase activity, indicating the presence of an unidentified HNF3β binding site which warrants more detailed investigation in the future.

In characterizing the recruitment of HNF3β to the promoter of CYP2B6 in a more physiologically relevant system, results from our ChIP assays in HPH confirmed the interaction between HNF3β protein and chromatin regions harboring the HNF3β-a and HNF3β-c motifs of *CYP2B6* gene. It is worth noting that HNF3β was also co-precipitated with the DNA fragment covering the -1.6/-1.4bp region, where an unidentified HNF3β binding motif was speculated. However, given the proximity between the -1.6/-1.4bp and the HNF3β-a containing (-1887/-1871bp) areas, such recruitment may only represent a HNF3β-a dependent phenomenon. Quantitative kinetics for binding affinities between HNF3β and the three predicted binding sites were further estimated by a SPR approach. Consistent with results of the luciferase reporter and ChIP assays, our SPR affinity assays revealed that the HNF3β-a and HNF3β-c elements were efficiently recruited to the HNF3β protein, while the HNF3β-b containing region appeared not to be directly associated with HNF3β.

In conclusion, our data show that HNF3β represents a novel LETF that regulates the transcription of *CYP2B6* gene, in addition to HNF4α and C/EBPα. Ectopic expression of HNF3β in HepG2 cells was associated with increased CYP2B6 transactivation and endogenous expression. Such enhancement can be at least partly explained by the identification and functional characterization of two consensus HNF3β binding sites located in the 5’-flank of CYP2B6 upstream. These findings reveal additional mechanistic bases in our understanding of *CYP2B6* gene transcription, and suggest that expression of CYP2B6 is governed by a complex regulatory network including genes such as, CAR, PXR, HNF4α, C/EBPα, and HNF3β. It is worth noting that multiple cis-acting responsive elements for C/EBPα and HNF4α have also been localized within the first 2 kb of the CYP2B6 promoter [12, 21]. Future studies to elucidate the mechanisms by which these LETFs work in concert with CAR/PXR to confer the optimal expression of CYP2B6 are warranted. Given that expression of HNF3β can be disturbed by the fluctuation of steroids and hormones, altered expression of HNF3β may contribute to the large interindividual variations in hepatic CYP2B6 expression.
